# Whole blood transfusion and paroxysmal nocturnal haemoglobinuria meet again: Minor incompatibility, major trouble

**DOI:** 10.1111/vox.13354

**Published:** 2022-09-14

**Authors:** Ingvild Jenssen Lægreid, Thomas Wilson, Kristoffer Hjertø Næss, Siw Leiknes Ernstsen, Vibeke Schou, Mirjana Grujic Arsenovic

**Affiliations:** ^1^ Department of Laboratory Medicine, Division of Diagnostic services University Hospital of North Norway Tromsø Norway; ^2^ Division of Prehospital services Finnmark Hospital Trust Kirkenes Norway; ^3^ Department of Anesthesia and Intensive Care Kirkenes Hospital, Finnmark Hospital Trust Kirkenes Norway

**Keywords:** blood components, haemolytic transfusion reaction, haemovigilance, transfusion reactions, transfusion therapy

## Abstract

**Background and Objectives:**

The field of transfusion medicine started out with whole blood transfusion to treat severe anaemia and other deficiencies, and then transitioned to component therapy, largely leaving the practice, and experiences, of whole blood transfusions behind. Currently, the field is circling back and whole blood is gaining ground as an alternative to massive transfusion protocols.

**Materials and Methods:**

Herein we describe a severely anaemic paroxysmal nocturnal haemoglobinuria (PNH) patient initially suspected of suffering from renal haemorrhage, receiving a standard low‐titre group O whole blood transfusion during pre‐hospital transportation.

**Results:**

Following the transfusion, the patient suffered a clinically unmistakable haemolytic transfusion reaction requiring supportive treatment in the intensive care unit. Clinical observations are consistent with an acute haemolytic reaction. The haemolysis was likely due to minor incompatibility between the plasma from the transfused whole blood and the patient's PNH red cells. Recovery was uneventful.

**Conclusion:**

This revealed an unappreciated contraindication to minor incompatible whole blood transfusion, and prompted a discussion on the distinction between whole blood and erythrocyte concentrates, the different indications for use and the importance of emphasizing these differences. It also calls attention to patient groups where minor incompatibility can be of major importance.

**Highlights**

- Low‐titre group O whole blood transfusion is not safe for all patients.
- Minor ABO incompatibility can be of major importance in patients with paroxysmal nocturnal haemoglobinuria.
- These issues should be addressed when transitioning from erythrocyte concentrates to whole blood in pre‐hospital emergency services.

## INTRODUCTION

During the 1970s and 1980s, civilian transfusion services transitioned from whole blood transfusion to component therapy; both to offer more tailored treatment for specific deficiencies and to better utilize donated units [1]. Component therapy has since been the rule, while the practice of whole blood transfusion has stood sanguinely by, before being picked up by military services. Leaning heavily on warzone experiences, whole blood transfusion is again on offer in some locations and its use is increasing.

Whole blood offers a convenient product when it comes to administration and is, at least theoretically, a more physiologically sound product with less dilutive additive solutions than a combination of erythrocytes, plasma and platelets. A recent report showed improved overall survival and decreased blood component use when whole blood is used in trauma patients [2], but meta‐analyses [3, 4] have failed to show superiority of whole blood compared to balanced massive transfusion. When excluding the logistical benefits, there is no definite evidence in favour of whole blood transfusion.

Transfusing group O whole blood results in non‐group O patients receiving incompatible plasma. To reduce the risk of haemolysis, low‐titre units are used. There is no clear definition of low titres, and centres offering low‐titre group O whole blood (LTOWB) report a range of accepted titres and techniques to obtain said titres [5]. In our region low titre is defined as anti‐A and anti‐B IgM and IgG titres <256. A study of patients receiving ≥4 units of whole blood comparing haemolytic markers between non‐group O and group O recipients, found no evidence of haemolysis [6], and LTOWB is considered safe for recipients of all ABO blood types.

However, as whole blood returns to transfusion practice, its potential benefits and risks for non‐trauma patients who might receive it should be evaluated, as we were reminded following one of the first whole blood transfusions in our region.

## CASE

A middle‐aged man with a history of aplastic anaemia, PNH and renal carcinoma presented to primary care complaining of extreme fatigue and generalized pain. Details of the patient's haematological medical history were unknown to the treating physician, but there was a note of previous admissions due to septicaemia.

Upon presentation, he was afebrile and clinically stable, with normal blood pressure (143/66 mmHg) and slightly elevated pulse rate of 91 bpm. The physician noted pallor, jaundice and dark urine. Haemoglobin was 4.6 g/dl, and the clinical suspicion was septicaemia and anaemia secondary to ongoing renal haemorrhage.

Due to long transportation distance, the patient was sent by air ambulance to the nearest hospital. As haemorrhage was suspected, he received one unit of LTOWB and tranexamic acid. He was clinically stable during transportation.

On arrival, he was still stable; blood pressure 184/89 mmHg, pulse 79 bpm and a slightly elevated respiratory rate of 20. Haemoglobin was 5.1 g/dl. Blood type was A RhD positive, antibody screen negative and direct antiglobulin test (DAT) positive. His on‐record blood type, however, was A RhD negative, and the reaction with anti‐D was explained by transfused O RhD positive whole blood. The blood bank noted dark plasma and suspected haemolysis. They contacted their transfusion consultant, who was aware of the patient's underlying haemolytic condition. The patient had suffered PNH‐related breakthrough haemolysis on several occasions, presenting with haemolytic anaemia requiring transfusion treatment. All prior antibody screens and DATs were negative and transfusion episodes uneventful. On admission the patient was stable, and taken together with the now recognized prior history and dark urine before the transfusion, the consultant found no immediate reason to suspect a reaction to the transfused blood.

However, unbeknownst to the blood bank, 1 h after admission the patient underwent rapid clinical deterioration with severe confusion, intense back pain, nausea, hypotension (systolic pressure 80 mmHg) and generalized skin erythema. He was transferred to the intensive care unit and treated with fluids, adrenaline, antihistamines, morphine and steroids for a suspected transfusion reaction. Haemoglobin had declined to 4.4 g/dl.

Haemolysis was confirmed by haemoglobinuria, dark plasma, elevated lactate dehydrogenase (LDH) and bilirubin, and low haptoglobin (Table 1). There were no values available from before the transfusion, and the contribution of each haemolytic process (underlying vs. acute) could not be established. As the blood bank was unaware of the reaction until the following day, the implicated product was no longer available, and a full transfusion reaction work‐up, including cross‐matching and cultures could not be completed. Both the patient and the donor were antibody screen negative, blood cultures negative and clinical observations are consistent with an acute haemolytic reaction.

**TABLE 1:** Relevant laboratory measurements during admission

	↓	Admission	Day 1	↓	Day 2	↓	Day 3	Day 5
Haemoglobin, g/dl (13–17)		5.1	4.1		5.7		8.5	8.4
Lactate dehydrogenase, U/L (105–205)		^a^	^a^		3174		2241	1285
Bilirubin, μmol/L (5–25)		57	47		26		24	‐
Creatinine, μmol/L (60–105)		155	159		125		111	94
Haptoglobin, g/L (0.3–2)		<0.3	‐		<0.3		‐	‐

*Note*: The admission values are *after* whole blood transfusion and *before* the clinical reaction. The initial clinical presentation is consistent with already ongoing haemolysis causing anaemia, and though the patient suffered a clinically obvious acute haemolytic transfusion reaction, the contribution of the whole blood transfusion on the laboratory parameters cannot be established. ↓ Denotes transfusion.

^a^ Denotes haemolysis interference.

After the acute treatment, the patient stabilized and eculizumab was infused followed by four units of A RhD negative erythrocyte concentrates. Haemoglobin level rose to 8.4 g/dl, bilirubin and creatinine normalized, while LDH decreased. Haptoglobin levels remained low throughout the 5‐day admission period.

Three months later, he again suffered from anaemia after PNH‐related breakthrough haemolysis requiring transfusion treatment, this time uneventfully with ABO‐identical erythrocyte concentrates. The antibody screen was still negative.

## DISCUSSION

PNH is an acquired haematopoietic disorder characterized by intravascular haemolysis, thrombosis and fatigue. It originates from clonal haematopoiesis, where a phosphatidylinositol glycan class A mutated clone gives rise to daughter cells having reduced expression of or lacking glycosylphosphatidylinositol‐anchored proteins. For red cells, this translates to a deficiency of complement regulatory proteins, most notably CD55 (decay‐accelerating factor) and CD59 (membrane inhibitor of reactive lysis) rendering them vulnerable to complement‐dependent haemolysis [7]. Before the introduction of complement inhibitors, treatment options were limited and haemolysis was an overwhelming problem. The resulting anaemia was treated by transfusion, and as haemolytic complications were frequently encountered [8], washed cellular products were recommended for many years [9, 10]. A retrospective study done on PNH patients transfused with various blood products over a 38‐year period concluded that washing is not necessary and that the use of type‐specific cellular products was safe [9], and currently no specific transfusion recommendations exist. Current literature addressing transfusion in PNH focuses on frequencies of red cell transfusion in the eculizumab era [11, 12].

Eculizumab, a C5‐inhibitor, has revolutionized PNH treatment. By C5 complement inhibition, membrane attack complex‐assembly followed by intravascular haemolysis is markedly decreased, resulting in reduced transfusion needs and improved quality of life for PNH patients [13]. Breakthrough haemolysis does occur, both intravascular at the end of the dosing interval, and extravascular due to C3‐deposition on the erythrocyte surface, yet overall eculizumab is an effective treatment [14]. The reduced need for transfusions, combined with the use of erythrocyte concentrates with minimal amounts of plasma and the protection offered by eculizumab, makes haemolytic transfusion reactions a rare event in this group.

For this patient, the latest flow cytometric quantification of PNH populations 10 months earlier showed 99% Type III cells among granulocytes and monocytes. As he was recently transfused at the time, erythrocytes were 65% Type III cells and 34% Type I cells. PNH‐population quantification was done by assessing the level of fluorescent aerolysin (FLAER) and CD24‐expression on CD15‐gated granulocytes, level of FLAER and CD14‐expression on CD64‐gated monocytes and level of CD59 expression on CD235a‐gated erythrocytes. He received treatment with biweekly eculizumab infusions. Unfortunately, before the described event, it was 3 weeks since the last dose. As whole blood was infused, donor‐derived anti‐A IgM, though in low titres, bound to the patient's erythrocytes initiating antibody‐mediated complement activation. Without the protection of complement inhibitors, natural or supplied, the patient's erythrocytes haemolysed, and the clinical description encapsulates a classic acute haemolytic transfusion reaction.

One might speculate that complement from the transfused whole blood contributed to the deterioration, as endogenous complement factors might have been at least partially consumed in the already ongoing haemolysis. Increasing levels of complement split products have been found in whole blood during storage [15], and on this note, there are other patient groups that probably should not receive plasma‐rich products. Patients with cold agglutinin disease have a steady state of depleted C3 and C4, and infusion of plasma could worsen haemolysis [16].

PNH cells are exquisitely sensitive to haemolysis, a property utilized in assessing haemolytic potential of alloantibodies and the effects of complement inhibition [17]. In an in vitro model using 2‐aminoethylisothiouronium treated cells to mimic PNH, investigators [18] showed the potential problems of minor incompatibilities in patients with complement inhibitory protein defects, thus making an in vitro prediction of the outcome of a minor incompatible whole blood transfusion to a PNH patient. As the haemolytic potential depends on clone size, patients with smaller PNH clones will probably not experience such a pronounced reaction, yet still present with biochemically evident haemolysis after transfusion.

As whole blood transfusion subsided and PNH patients receive treatment offering protection from complement‐mediated haemolysis, thus reducing transfusion needs, knowledge of well‐known complications when transfusing PNH patients with out‐of‐group plasma‐rich products is evanescing. We were reminded as one of the first pre‐hospital whole blood transfusions was given to a sub‐optimally treated PNH patient. Non‐group O PNH patients now have a note in the regional transfusion information technology (IT) system to avoid transfusions resulting in minor incompatibility.

This illustrates the need to emphasize that whole blood and erythrocyte concentrates are not interchangeable products for all patients when transitioning from one to the other in pre‐hospital services. One must call attention to the difference plasma makes and show restraint in transfusing anaemic non‐bleeding patients with whole blood.

## CONFLICT OF INTEREST

The authors disclose no conflicts of interest.
